# Enhancing Brain Health Promotion and Dementia Detection in Primary Care with the KAER Framework

**DOI:** 10.1093/geroni/igaf122.4247

**Published:** 2025-12-31

**Authors:** Lisa McGuire, Jennifer Pettis

**Affiliations:** Gerontological Society of America, Atlanta, Georgia, United States; Gerontological Society of America, Washington, District of Columbia, United States

## Abstract

The Gerontological Society of America’s (GSA) KAER Toolkit for Brain Health is intended to support primary care teams in implementing a comprehensive approach to initiating conversations about brain health, detecting and diagnosing dementia, and providing individuals with community-based supports. Based on the four steps of the KAER framework—Kickstart, Assess, Evaluate, and Refer (KAER)— the Toolkit includes practical approaches, educational resources, and validated clinical tools that teams can immediately integrate into their workflow. Recently, GSA launched the third version of the publicly available GSA KAER Toolkit that was revised based on expert review and evaluation of the latest scientific evidence for its clinical applicability. This online version allows for frequent updates aligning with the ever-evolving brain health scientific-landscape, including significant advances in brain health promotion, dementia risk reduction, advances in biomarkers, and recent guideline updates on use of screening and detection tools. Attendees at this session will learn about the flexible KAER framework and how it can be integrated into existing workflows within their practice setting through the entire diagnostic process of K to A to E and to R improve care and outcomes of older adults living with dementia and their caregivers. This session will highlight newly added resources to the KAER Toolkit including brain health promotion, dementia risk reduction, common behavioral and psychological symptoms association with dementia, and updated screening and diagnostic tools. Additionally, practice pearls about how aspects of the KAER toolkit have been integrated into various clinical practices will be discussed.

